# The characteristics of dome-shaped macula in Chinese children aged 4–6 years using optical coherence tomography angiography

**DOI:** 10.1186/s12886-022-02630-5

**Published:** 2023-01-25

**Authors:** Lu Xiang, Yingming Zhou, Xuan Zhang, Kai Li, Chunli Fei, Yangyang Wang, Yang Bai, Bing Xie, Xi Shen

**Affiliations:** 1 Department of Ophthalmology, Ruijin Hospital, Shanghai Jiaotong University of Medicine, No,197, Ruijin 2nd Road, Huangpu District, Shanghai, China; 2 Department of Ophthalmology, Wuxi Branch of Ruijin Hospital, Shanghai Jiaotong University of Medicine, Shanghai, China

**Keywords:** Dome-shaped macula, Chinese, Preschool children, Optical coherence tomography angiography

## Abstract

**Purpose:**

To evaluate the characteristics of dome-shaped macula (DSM) in children aged 4–6 years with normal visual acuity using optical coherence tomography angiography.

**Method:**

This is a cross-sectional study. A total of 19 children aged 4–6 years were included. The results of optical coherence tomography angiography images were analysed to identify and quantify retinal structural and vascular parameters in DSM children. The dome height, dome base, and sub-dome choroidal thickness were manually measured. Participants with DSM and those without DSM from our previous study were compared on these parameters.

**Results:**

Nineteen eyes of the preschool subjects with normal visual acuity showed horizontal DSM on optical coherence tomography (OCT). The DSM was significantly smooth and low in the children, and we did not observe differences between sex and age. Compared to the children without DSM, the average axial length was longer, and the average macular vessel density was lower in the DSM group, especially in the deep retinal vascular density. Additionally, the dome height was positively correlated with the sub-dome choroidal thickness. When the dome base/height was increased, the fovea avascular zone (FAZ) area was larger.

**Conclusion:**

Dome-shaped macula was detected in the preschool children in the process of the emmetropization with normal visual acuity. The changes in macular structure and vasculature provide new ideas for further investigation into the characteristics of DSM formation.

## Translational Relavance:

Dome-shaped macula may be an important biomarker for problems in visual acuity, particularly myopia, long before the patients become symptomatic in preschool children.

## Introduction

The dome-shaped macula (DSM) is a morphological change, characterized by a macular bulge with a height of more than 50 μm. It was first proposed in high myopia with posterior staphyloma by Gaucher in 2008 [1]. OCT is widely used in the diagnosis of retinal diseases [2], [3], [4], [5], which provides the basis for the diagnosis of DSM. Many studies have attempted to explore the causes and complications of these changes [6], [7], [8], [9], [10], [11], [12]. Gaucher et al. [1] first assumed the hypothesis as localised choroidal thickening and resistance to posterior staphylomatous deformation. Mehdizadeh et al. [11] inferred that ocular hypotony and tangential vitreomacular traction cause DSM. Imamura et al. [12] speculated that the DSM was potentially caused by a local thickening of the subfoveal sclera. However, these studies were conducted on highly myopic eyes, and these hypotheses are also influenced by the anatomical changes related to the high myopia. According to related studies, DSM does not occur only in high myopic eyes, but also in mild to moderately myopic eyes [10], even in emmetropic eyes [13]. Thus, the given mechanisms may not fully explain the pathophysiology of DSM.

In addition, reports show that elder subjects usually showed bidirectional domes, whereas young subjects have unidirectional domes. It remains unclear whether these have a common aetiology [10]. Previous DSM studies have focused mainly on adults [1], [12], [14], [15], [16], [17], but only few of them focused on children [18], [19], [20]. The presence of DSM has not been verified by existing studies that used OCTA to assess preschool children. Clarifying the structural and vascular characteristics of DSM in children without complications in the posterior pole could provide clues on its intrinsic cause.

Normal retinal structure and vasculature are prerequisites to meeting the normal retinal metabolic requirements and forming the normal visual function in preschool children [21], [22]. Some studies have revealed that the structural and microvascular changes predate the onset of symptoms in retinal and optic disc diseases [4], [23], [24], [25]. Thus, we aimed to determine whether it is possible to detect deviations in the retinal thickness and vasculature in children with asymptomatic DSM, and whether the deviations are correlated with the DSM related parameters and the cause of DSM.

Thus, the current study evaluated the structural and vascular characteristics of DSM and compared these with eyes without DSM using OCTA in the Chinese preschool children to determine the potential cause of DSM.

## Materials and methods

The institutional review board and ethics committee of the Ruijin Hospital in Shanghai, the People’s Republic of China approved this retrospective study, which was adhered to the tenets of the Declaration of Helsinki. We obtained the written informed consent from the parents or guardians before the retrieval of records.

**Fig. 1:**
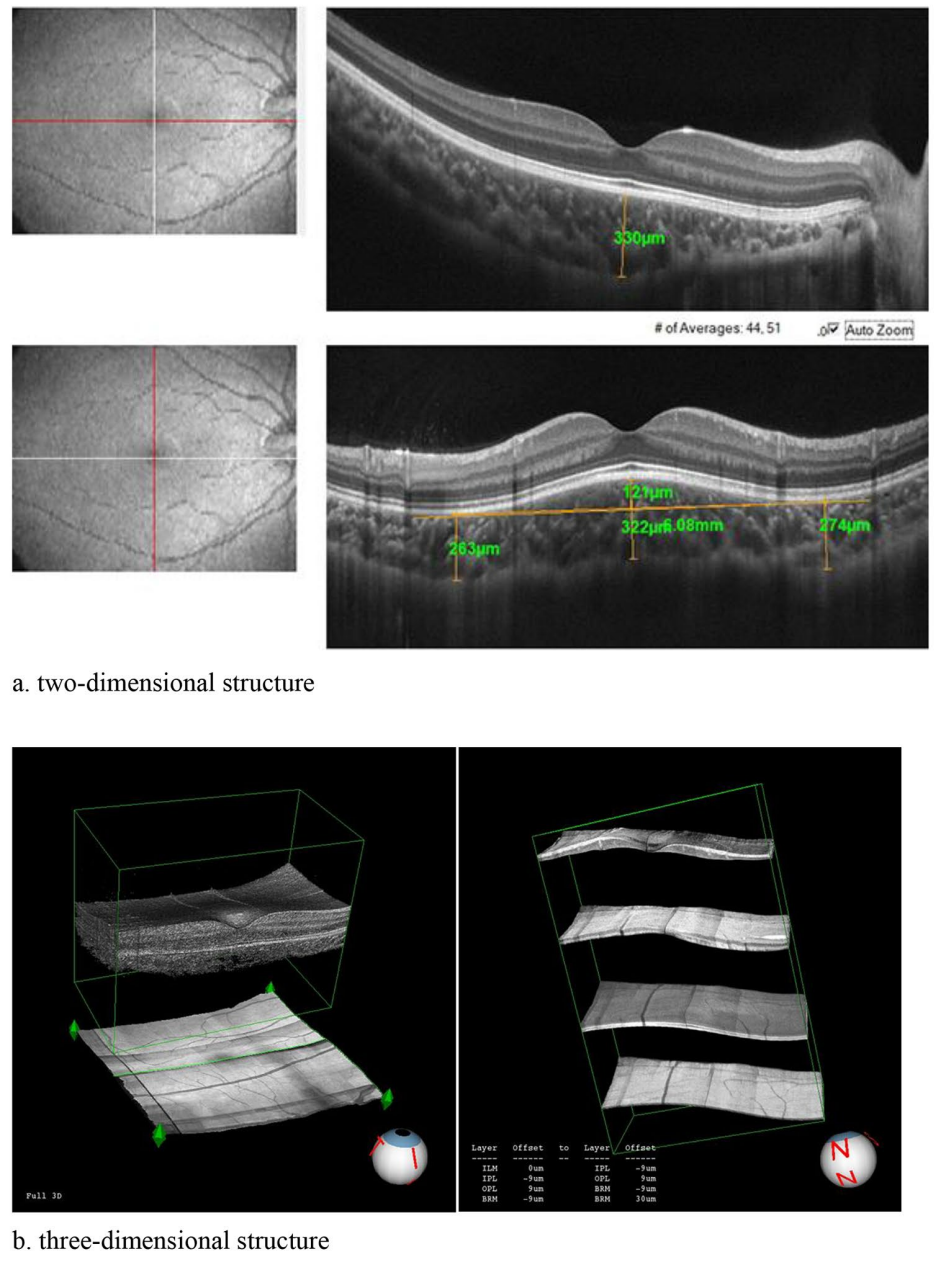
Schematic diagram of the two and three-dimensional structure of the dome shaped macula

We examined 343 healthy Chinese subjects aged 4–6 years old and enrolled 19 healthy children with DSM (a.k.a. the DSM group) between June 2020 and March 2021 (Fig. 1). In addition, we have assessed the anterior segment and retinal parameters of healthy Chinese preschool children aged 4–6 years in a previous study [26], also called normal group. All these children underwent a comprehensive ophthalmic examination, including slit-lamp biomicroscopy, measurement of best-corrected visual acuity (BCVA), autorefraction, non-contact intraocular pressure, and optical biometry scans (axial length, anterior chamber depth, central corneal thickness, and crystalline lens thickness). Atropine was instilled in both eyes three times a day for three consecutive days before data collection to facilitate cycloplegic refraction and fundoscopy. The following were included: (1) subjects with available OCTA images showing the dome-shaped macula; (2) spherical equivalent between + 1.0 and + 2.5D in 4-year-old child, between + 0.5 and + 2.0D in 5- and 6-year-old child, astigmatism less than 1.0 D; (3) non-contact intraocular pressure less than 21mmHg. Exclusion criteria included children with pathological anisometropia (spherical difference more than 1.50D), eye surgery history, amblyopia, nystagmus, retinal disease, inflammation, prematurity, neurologic disease, or systemic conditions in the ophthalmic examinations.

DSM was defined as an elevation of the macular retinal pigment epithelium (RPE) and Bruch membrane (BM) of > 50 μm on the vertical and/or horizontal scans of the OCT images [1]. With the definition in the research of Caillaux et al. [15], we classified DSMs into three patterns: DSMs present in the vertical scans, but not in the horizontal scans, called horizontal oval-shaped dome (horizontal DSM); DSMs present only in the horizontal scans, called vertical oval-shaped dome (vertical DSM); and DSMs present both in the vertical and horizontal scans, called round dome. We further manually measured the dome height, dome base, and sub-dome choroidal thickness in the scans showing the elevation. The dome height was defined as the distance between the peak of the bulge and a line running parallel to the RPE at the base of the bulge. The dome base was defined as the distance between two lowest point at the base of the elevation. The choroidal thickness was defined as the distance between the outer border of the RPE line and the chorioscleral interface. The sub-dome (SDCT) was measured at the peak of the bulge. All of these DSM related parameters were manually measured by two independent examiners. The asymmetry index (AI) was used to assess the asymmetry between the superior and inferior hemiretina, which was used in the early diagnosis of myopia and glaucoma [27], [28]. The equation AI= |log10 (superior/inferior hemiretinal thickness) | was used to calculate the asymmetry indices. We evaluated the correlations between the AI and DSM related parameters. Horizontal and vertical OCTA scans centred at the fovea were performed using the RTVue Avanti spectral-domain optical coherence tomography equipped with AngioVue software (Version 2017,1,0,155; Optovue, Inc., Fremont, CA, USA) in the high-definition (HD) disc scan ($4.5\times 4.5\text{m}\text{m}$) and HD retina scan ($6.0\times 6.0\text{m}\text{m}$) modes. An experienced examiner who was blinded to the subjects’ information performed the OCTA assessment. The segmentation of the retinal structural and vascular layers was described in previous studies [29], [30], [31], [32], [33]. The magnification effect of the structural and vascular parameters in the retina was corrected by the Littman formula and the modified Bennett formula [34]. Both eyes of all subjects were examined, if both eyes met the enrollment conditions, one eye was randomly selected for inclusion.

Statistical analysis was performed by a commercially available statistical software package (SPSS for Mac, version 22.0; IBM SPSS, Inc., Chicago, IL, USA). We calculated the descriptive statistics using means, standard deviations (SD), and percentages. A Shapiro-Wilk test was applied,confirming that all continuous variables were normally distributed. The independent sample t-test was used to distinguish the differences between sex or eye-type, and one-way ANOVA was used to assess differences among ages in the DSM group. The independent one-sample t-test was used to compare the AL, anterior segment, and retinal related parameters between the DSM group and normal group, and the Pearson correlation analysis was used to assess a correlation between the DSM related parameters (such as dome height, dome base, and SDCT) and the retinal parameters. The intraclass correlation coefficients (ICC) were used to assess the agreement of two measurement of DSM related parameters. All P values were 2-sided and considered to be statistically significant if their values were less than 0.05.

## Results

This cross-sectional study included 19 eyes of 19 children (10 female, 9 male) with DSM as the DSM group. And 242 eyes of 242 children with normal fundus morphology from our previous study form the normal group. The anterior segment parameters (Table 1) and retinal-related parameters (Tables 2 and 3) of the two groups were compared. In addition, the DSM group will be analysed for correlation between segment and retina-related parameters and DSM-related parameters. The ICC was greater than 0.8 in the two measurements of the DSM related parameter, and the final values were the average values of the two measurements.

**Table 1:** Comparison of ocular parameters and demographic data between 4-6-year-old Chinese preschool DSM and normal children

Variables	DSM group	Normal group	P value
Male/Female	10/9	126/116	—
Age(year)	4.89 ± 0.46	5.31 ± 0.74	—
Axial length(mm)	22.61 ± 0.81	22.37 ± 0.71	0.167
Central corneal thickness(µm)	553.16 ± 22.39	535.96 ± 28.79	0.012
Anterior depth(mm)	2.87 ± 0.31	2.80 ± 0.25	0.220
Lens thickness(mm)	3.64 ± 0.10	3.70 ± 0.20	0.190

DSM: dome shaped macula; Data are mean ± standard deviation (SD)

**Table 2:** Comparison of retinal thickness between 4-6-year-old Chinese preschool DSM and normal children

	DSM group		Normal group		P value	
	IRT(µm)	FRT(µm)	IRT(µm)	FRT(µm)	IRT	FRT
average	100.08 ± 6.92	287.52 ± 12.84	98.99 ± 5.66	286.13 ± 11.52	0.427	0.617
fovea	51.52 ± 9.51	232.05 ± 19.27	49.29 ± 8.82	228.75 ± 17.68	0.294	0.436
parafovea	105.25 ± 5.71	308.54 ± 10.15	104.38 ± 7.03	309.36 ± 12.62	0.599	0.783
temporal	102.76 ± 11.76	301.32 ± 11.43	97.92 ± 5.84	300.72 ± 12.96	0.002	0.844
superior	104.67 ± 8.72	311.04 ± 12.59	107.33 ± 8.26	315.12 ± 13.09	0.180	0.191
nasal	105.33 ± 5.41	310.33 ± 15.14	104.75 ± 7.57	312.56 ± 13.35	0.745	0.488
inferior	108.27 ± 8.09	311.47 ± 14.96	107.53 ± 8.39	309.04 ± 13.10	0.708	0.442
perifovea	100.35 ± 9.30	282.63 ± 14.74	99.26 ± 6.03	281.32 ± 11.95	0.469	0.651
temporal	89.91 ± 12.01	267.09 ± 11.55	87.60 ± 6.43	268.66 ± 12.63	0.165	0.601
superior	96.87 ± 9.49	285.34 ± 11.76	90.02 ± 8.43	285.66 ± 12.55	0.291	0.913
nasal	114.22 ± 6.65	299.54 ± 10.87	113.93 ± 6.40	299.04 ± 13.10	0.854	0.870
inferior	97.72 ± 7.17	270.56 ± 10.09	96.53 ± 8.21	271.79 ± 12.44	0.540	0.675

IRT: inner retinal thickness; FRT: full retinal thickness; DSM: dome shaped macula

Data are mean ± standard deviation (SD)

**Table 3:** Comparison of retinal vasculature between 4-6-year-old Chinese preschool DSM and normal children

	DSM group		Normal group		P value	
	SCP(%)	DCP(%)	SCP(%)	DCP(%)	SCP	DCP
average	46.89 ± 4.20	44.30 ± 7.63	48.09 ± 2.92	48.74 ± 6.51	0.095	0.005
fovea	20.12 ± 7.45	33.45 ± 9.28	21.16 ± 6.65	33.15 ± 7.99	0.515	0.877
parafovea	48.49 ± 6.15	47.86 ± 8.97	49.94 ± 3.79	51.93 ± 6.10	0.129	0.008
temporal	49.31 ± 6.12	49.97 ± 8.54	49.93 ± 4.26	52.62 ± 6.09	0.552	0.079
superior	49.19 ± 7.00	47.72 ± 7.57	50.98 ± 4.13	51.39 ± 7.00	0.087	0.029
nasal	47.16 ± 4.25	49.33 ± 8.87	49.14 ± 4.26	52.88 ± 6.15	0.052	0.020
inferior	48.31 ± 9.65	44.40 ± 12.29	49.68 ± 4.73	50.82 ± 6.97	0.271	< 0.001
perifovea	47.66 ± 4.35	45.08 ± 7.98	48.90 ± 2.91	48.66 ± 7.08	0.087	0.037
temporal	44.99 ± 3.82	47.28 ± 8.90	46.25 ± 3.73	49.97 ± 6.83	0.161	0.107
superior	48.93 ± 4.26	45.61 ± 8.84	49.10 ± 3.12	48.76 ± 7.77	0.824	0.093
nasal	50.32 ± 5.11	44.63 ± 7.11	51.35 ± 3.09	48.40 ± 7.50	0.186	0.035
inferior	47.61 ± 4.33	43.85 ± 7.73	48.84 ± 3.52	47.48 ± 8.01	0.149	0.057

SCP: superficial retinal vascular density; DCP: deep retinal vascular density; DSM: dome shaped macula; Data are mean ± standard deviation (SD)

**Fig. 2:**
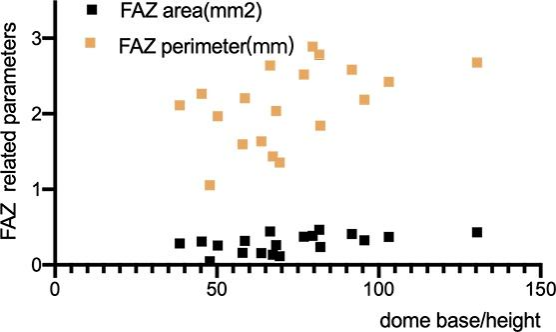
Correlation between the dome base/height and the FAZ related parameter

**Fig. 3:**
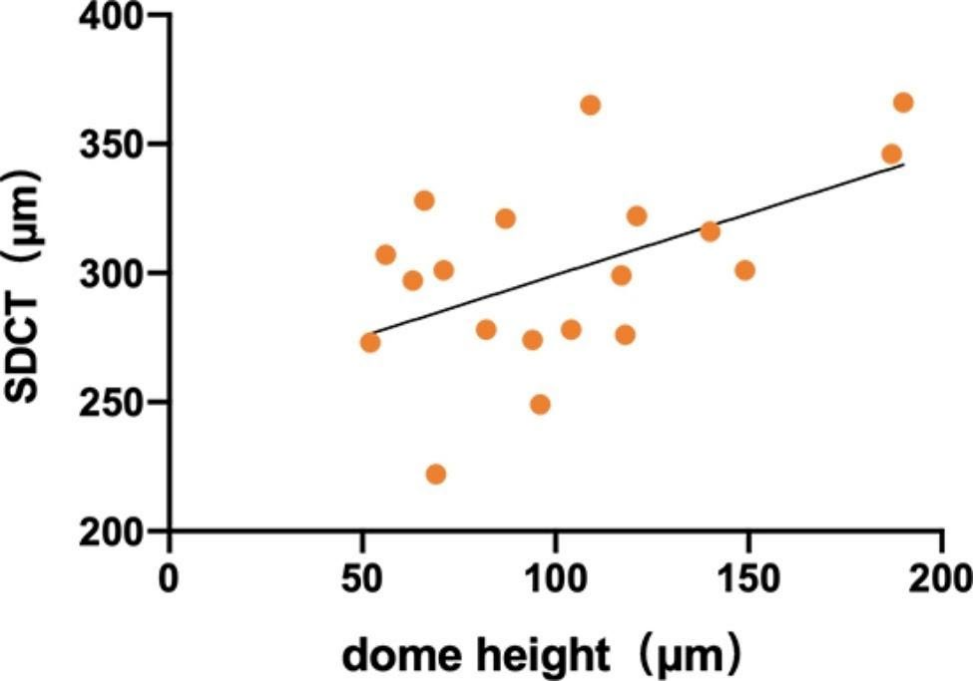
Correlation between the dome height and the SDCT

No statistically significant age or sex differences were found in DSM-related parameters. In the DSM group, the inner retinal thickness was positively correlated with the superficial retinal vascular density (r = 0.602, P = 0.006). The foveal thickness and vessel density were negatively correlated with the FAZ area (r=-0.548, P = 0.015; r=-0.757, P < 0.001). We further explored the associations between the other ophthalmic parameters and DSM-related parameters and found that the FAZ area and perimeter were both positively correlated with dome base/height (correlation coefficient = 0.505, P = 0.027; correlation coefficient = 0.467, P = 0.044, Fig. 2). In addition, the full retinal thickness was negatively correlated with SDCT (correlation coefficient=-0.600, P = 0.007). The dome base was positively correlated with dome height (correlation coefficient = 0.605, P = 0.006) and SDCT (correlation coefficient = 0.558, P = 0.013). The dome height was also positively correlated with SDCT (correlation coefficient = 0.524, P = 0.021, Fig. 3). Besides, we explored the correlations between the AI and DSM-related parameters and found no correlations.

Compared with normal group, eyes with DSM had thicker central corneal thickness (553.16 ± 22.39 μm vs. 535.96 ± 28.79 μm, P < 0.05) and longer axial length (22.61 ± 0.81 mm vs. 22.37 ± 0.71 mm, P = 0.167). The mean inner and full retinal thickness were thicker in the DSM group than in the normal group, except that the full retinal thickness in the parafovea was thinner in the DSM group. No significant differences in retinal thickness of each quadrant were identified between the two groups. Table 2 lists the retinal vascular differences between two groups, and we found that the deep retinal vascular density was lower in the DSM group than in the normal group, in almost every quadrant except in the fovea (33.45 ± 9.28% vs. 33.15 ± 7.99%).

## Discussion

In the current study, the DSMs in the Chinese children aged 4–6 years were detected only in the vertical OCT sections, which was similar to the findings in the previous study of young subjects [10]. To date, we know of no study with such young subjects with hyperopia. Our observations showed that the DSMs could present in young hyperopes with normal visual acuity.

It may be the earlier stage of DSMs which showed smooth elevation with a broad dome base and a low dome height, compared to the previous studies in elder patients [6], [15], [35]. Xu et al. [10] reported that the macular elevations only in one meridian across the fovea without the spatial association could be called ridge-shaped macula. These were found in highly myopic patients. The current study showed the macular elevations in hyperopic eyes lack the characteristics in myopic eyes but they are still labeled as DSM. And it would be helpful to investigate the cause of DSM according to the current features at a clinically asymptomatic stage. Zhao et al. [36] measured the choroidal thickness at the top and the base of the dome and classified the choroidal thickness in DSM into three distinct patterns. The sub-dome choroidal deepening was defined as a choroid at the sub-dome that was thicker by a factor of 1.5 as compared to that at the peri-dome. The peri-dome choroidal deepening was defined as the ratio of less than 0.67. The absence of choroidal deepening was between 0.67 and 1.5. Different patterns might represent different cause of DSM formation [36], and we only detected the absence of choroidal deepening in the current study.

Previous studies have shown that DSM formation is due to local choroidal thickening in the macula [1], and other studies have noted that high myopia with DSM formation exhibited local uneven changes in the sclera [12]. Our study found a positive correlation between SDCT and the dome height, thus we speculated the inward pushing of the choroid at the sub-dome could be the cause of DSM. Besides, the choroid plays a compensatory role in the process of emmetropization, that is, the choroid thickens and the retina moves forward in the case of myopic defocus, and the choroid thins and the retina moves backward in the case of hyperopic defocus [37]. Therefore, we speculate that the DSM formation during the emmetropization stage of children may be due to the compensatory effect of the sub-dome choroid.

We found that compared with normal children, the central corneal thickness was thicker, axial lengths were longer, and superficial and deep retinal vascular density were lower in the DSM group. However, the foveal deep retinal vascular density in the DSM was significantly higher than that in normal children. In children with hyperopia, a thick, flat cornea protects against myopia [38]. In the presence of retinal abnormalities, deep retinal vascular density has been reduced even in the absence of clinical symptoms [39]. We speculated that during the process of emmetropization in children, the axial length of the eye became excessively elongated, and the deep retinal vascular density began to decline. However, the appearance of DSM plays a compensatory role in the fovea vasculature, and the vascular density in this area is slightly increased compared to normal children. In addition, we found that both the inner and full retinal thickness were increased in DSM group, but the difference was statistically significant. Other studies have speculated that the folding of Bruch’s membrane is the initiating factor of DSM, pushing the retina to the inner layer and reducing the thickness of the retina [40], which is contrary to our findings. This inconsistency may be due to differences in age and ethnicity of our study population, or perhaps different initiating factors at different stages of DSM.

Existing studies have found that the FAZ area was decreased in retinopathy of prematurity, amblyopia, etc. [41], [42]. Other studies found that the FAZ area was not correlated with age in preschool children and adolescents [43], [44], [45]. However, we found that steeper DSMs correlated with greater decrease in the FAZ area. The FAZ area in normal and DSM groups were not significantly different. It showed that the DSM might do no harm to visual acuity in the early stage and with a relatively normal FAZ area. However, if the dome height continued to increase to a certain height, it might have an influence both in the FAZ area and foveal vasculature. Our study recommends long-term and close follow-up monitoring of visual acuity, refraction, and fundus changes in children with DSM.

Numerous OCT imaging studies have described morphological alterations in DSM [13], [15], [46], but few have quantified the alterations in the retinal layers. We speculate that the uneven inward growth of the posterior pole during the process of emmetropization may lead to uneven changes in the retinal layers. The AI was used to assess the symmetry in the superior and inferior retina. As expected, the retina was symmetry in normal eyes; the nasal hemiretina was thicker than the temporal hemiretina in the normal eyes, the change of asymmetry between the nasal and temporal hemiretinas cannot be simply described by AI [27]. However, we have not found correlations between AI and DSM-related parameters. The retina at the posterior pole is relatively symmetrical with respect to the superior and inferior hemiretinas. The AI was initially used to aid in the diagnosis of patients with early glaucoma and was later used to monitor irregular expansion of the posterior pole in children with myopia [27], [28]. However, the dome shaped macula only occurred in the horizontal direction in our study, which may be the characteristic of DSM in children and at the early stage, thus no obvious changes in retinal symmetry in the posterior pole were found. In the elderly and in advanced DSM, AI may be used to monitor the changes in retinal symmetry and to explain the cause of DSM.

Finally, we found increased axial length and decreased macular vascular density in children with DSM. Our previous findings show that children with myopia exhibited changes in choroidal thickness prior to evident changes in retinal thickness [47] and they also showed increased AI. In addition, the existing studies found a higher incidence of DSM in the myopic population, especially in patients with high myopia [14], [48], [49]. Therefore, we believe that the detection of DSM in childhood with regular examination of axial length, vascular density, choroidal thickness, and changes in AI may play a vital role in the early prediction of myopia to some extent.

There are several limitations in this study. First, the number of eyes was relatively small and the sample may not be representative. Second, cross-sectional studies have rarely demonstrated causality, and the cause of DSM can only be speculated. In addition, due to the limitation of equipment, our study can only measure up to the choroid. Future studies could increase the scanning range and deepen the scanning depth to identify more factors. Nonetheless, we have not found any similar studies on DSM in preschool children, and our investigation was the first to explore both the structural and vascular characteristics of the retina and choroid in DSM.

In conclusion, we detected the horizontal DSM in the 4-6-year-old preschool children. The appearance of DSM may be a compensatory response to excessive elongation of axial length during emmetropization, and the early stages of DSM may be associated with local thickening of the choroid. DSM is expected to become a marker in preschool children, prompting long-term monitoring of visual acuity, axial length, and diopter.

## Data Availability

The data that support the findings of this study are available from the corresponding author, XS, upon reasonable request.
